# The quest for equity in Latin America: a comparative analysis of the health care reforms in Brazil and Colombia

**DOI:** 10.1186/1475-9276-11-6

**Published:** 2012-02-02

**Authors:** Roberto JF Esteves

**Affiliations:** 1 Secretariat of Labor and Education Management for Health (SGTES), Ministry of Health of Brazil, Esplanada dos Ministérios, Bloco G, Ed. Sede, Sala 704, Brasília, DF, 70680-350, Brazil

**Keywords:** Brazil, Colombia, health care reform, health care system, equity, health inequities, comparative analysis, health policy

## Abstract

**Introduction:**

Brazil and Colombia have pursued extensive reforms of their health care systems in the last couple of decades. The purported goals of such reforms were to improve access, increase efficiency and reduce health inequities. Notwithstanding their common goals, each country sought a very different pathway to achieve them. While Brazil attempted to reestablish a greater level of State control through a public national health system, Colombia embraced market competition under an employer-based social insurance scheme. This work thus aims to shed some light onto why they pursued divergent strategies and what that has meant in terms of health outcomes.

**Methods:**

A critical review of the literature concerning equity frameworks, as well as the health care reforms in Brazil and Colombia was conducted. Then, the shortfall inequality values of crude mortality rate, infant mortality rate, under-five mortality rate, and life expectancy for the period 1960-2005 were calculated for both countries. Subsequently, bivariate and multivariate linear regression analyses were performed and controlled for possibly confounding factors.

**Results:**

When controlling for the underlying historical time trend, both countries appear to have experienced a deceleration of the pace of improvements in the years following the reforms, for all the variables analyzed. In the case of Colombia, some of the previous gains in under-five mortality rate and crude mortality rate were, in fact, reversed.

**Conclusions:**

Neither reform seems to have had a decisive positive impact on the health outcomes analyzed for the defined time period of this research. This, in turn, may be a consequence of both internal characteristics of the respective reforms and external factors beyond the direct control of health reformers. Among the internal characteristics: underfunding, unbridled decentralization and inequitable access to care seem to have been the main constraints. Conversely, international economic adversities, high levels of rural and urban violence, along with entrenched income inequalities seem to have accounted for the highest burden among external factors.

## Introduction

### Background

Latin America is a vast, heterogeneous land, composed of a multitude of cultures and traditions. Nevertheless, a common heritage is sadly persistent: entrenched social and health inequalities. While in 2005 a child in Cuba had a better chance of surviving to age 5 than one living in the United States, a child from Haiti had a worse chance of reaching the same age than one from Namibia [1]. On the other hand, while someone born in 2005 in Costa Rica could expect to live longer than someone born in Denmark that same year, another one born in Bolivia could hardly hope to live as long as someone born in Kazakhstan [1].

Colombia and Brazil, the two most populous countries of South America, face similar struggles within their own borders. Bearing the largest Gini coefficients of the region: 0.564 (Brazil, 2005) vs. 0.5849 (Colombia, 2006), their political leaders decided in the late 1980s and early 1990s to attack some of the underlying causes of those disparities. Many social policies were then instituted. Among them, two of the most profound and far reaching were the reforms of their health care systems. Remarkably, despite the fact that such reforms were initially pursued just 5 years apart from one another, each country ventured in almost opposite directions. While Brazil sought to reestablish a greater level of State control through a public national health system, Colombia embraced the philosophies of employer-based social insurance and market competition.

The present study thus aims to shed some light onto why they pursued different pathways and what that has meant in terms of health outcomes. Some of the questions addressed by this work include: *A) How effective have these reforms been in reducing health inequities? B) How do these reforms compare to one another? C) What lessons can be learned from the respective reform processes?*

### Equity Frameworks

Health inequities have been the subject of a thriving debate in the last few decades, drawing contributions from philosophers, economists, social scientists and physicians alike. Despite the variety of perspectives, they all seem to stem from the empirical observation that while the world is now materially richer than in any other point in known history, thousands continue to suffer and die every day due to preventable and treatable diseases. Furthermore, the widening gap in health status between developed countries and developing countries, as well as between the rich and poor segments of societies within many countries, seems to invoke a sense that something is wrong.

Spurred by such troubling thoughts, several bodies of theories have emerged. Most of them can be grouped into five categories: utilitarian approaches, communitarian theories, egalitarian theories, libertarian (market-based) approaches, and deliberative democratic procedures. Ranging the gamut from consequentialist (concerned with outcomes) to proceduralistic (concerned with the process), each set of approaches has its strengths and weaknesses. In summary, utilitarian theories of health care justice require that resources be allocated in order to maximize net social utility. It is not concerned with individual inequities as long as society as a whole is better off. Communitarian theories express that there are no universal norms of social justice, but rather that those are constructed by each society through a process of social and political evolution. Under this framework, if a given society values other goals higher than health, then it has no overarching responsibility to secure it for its members. Egalitarian theories embed two different approaches: one sees that everyone is entitled to the same level of health achievement while the other believes that everyone is entitle to equal opportunities of achieving good health. While the former has a value preference for the outcomes, the latter values more the means to achieve them. Libertarian perspectives take a more blatant position: society has simply no obligation to address social or health inequalities because any measure to do so would imply redistributive policies that ultimately infringe on individual liberties [2]. Therefore, providing for one's health is an individual responsibility rather than a societal obligation. Deliberative democratic procedures are defended by those who believe that by espousing the principles of autonomy, political equality and due deliberation within an open public process, justice will prevail. However, they offer little guidance over what principles of justice should take precedence over others, if any.

As interesting an exercise as this would be, it is beyond the scope of the present work to further dissect the different ethical perspectives. For the motivated reader who would like to do so, the thorough review presented in the first chapter of "Health and Social Justice" [3] is an enlightening journey. Nevertheless, before we proceed any further, some key issues should be clarified.

First, it is important to distinguish between inequalities and inequities. Are all health inequalities considered inequitable? Not necessarily, unless they can be considered *avoidable, unnecessary *and *unfair *[4]. For instance, the observed *higher *life expectancy for women compared to men in developed countries cannot be construed as inequitable if it is due to *intrinsic biologic differences *that are beyond the reach of current social or medical interventions to overcome it [5]. Conversely, the observed *lower *life expectancy for women in many developing countries can be deemed inequitable if is due to *social policies *that discriminate against women. Such difference is not only inherently unfair, but also avoidable and unnecessary, for if both men and women could equally benefit from societal resources, their life expectancies would tend to be reversed, as seen in the developed world.

Second, although it is widely recognized that social inequities have direct and indirect impacts on health [6], dealing with them alone is unlikely to produce the highest attainable standard of health defined in article 12 of the International Covenant on Economic, Social and Cultural Rights [7]. Moreover, the existence of a universal health care system, based on fair financing mechanisms and quality delivery systems, is seen as an essential tool to tackle the social determinants of health [8].

Third, the false dichotomy pointed out earlier between consequentialist and proceduralistic approaches of justice in health hinders any comprehensive understanding of the complex nature of health equity. Conceptually, health equity is a multifaceted praxis. It includes concerns about achievement of health and the capability to achieve good health [9], not just one *or *the other. Fortunately, a novel approach has recently emerged as an attempt to bridge this gap: the *health capability paradigm*. Developed by Jennifer Ruger [10], it draws its core elements from Amartya Sen's capability framework [11] but attempts to further specify it to the health field and provide mechanisms to make it operational.

In essence, the health capability paradigm sees *human flourishing *- an Aristotelian concept of *good life *- as the ultimate human goal. In order to achieve it, one needs to be able to enjoy some basic capabilities. Ruger argues that health is one such critical component. Likewise, health capability entails two essential components: *health functioning *and *health agency*. Health functioning can be understood as the medical construct of "health" (physical and mental well-being), while health agency can be expressed as the "ability to control personal and professional situations to pursue health." Thus, health capabilities represent an individual's ability to achieve certain health-related functionings and the freedom to achieve them [3].

Universal health insurance is paramount to the health capability paradigm. It requires that the health system ensures access to medically necessary and medically appropriate care. Furthermore, it states that health care resources should be distributed solely on those criteria. As a result, any discrimination based on the ability to pay, gender or ethnicity is unacceptable. Thus, the central ethical aims of universal health insurance coverage are to make and keep people healthy, to develop their health functioning and health agency, as well as enhance their security by protecting them from the physical and economic consequences of ill health. This is not to say that every health intervention must be offered within this perspective. On the contrary, only those that satisfy the above medical criteria, and are of high quality, would be deemed eligible. In addition, when resources are scarce, as it is almost always the case (especially in the developing world), preference should be given to central health capabilities as opposed to non-central ones (e.g. life-saving interventions vs. cosmetic surgical procedures). Besides, the design of any package of benefits should be sought through a scientific and deliberative process that includes individuals, physicians and public health experts in an attempt to reach a reasoned consensus. Such deliberations will facilitate the development of health policy within an institutional arrangement of shared health governance, a *modus operandi *in which individuals, providers and institutions work together to empower individuals and create an environment enabling all to be healthy [3].

Finally, given the breath of possible approaches inherent in the application of the health capability paradigm to the analysis of any national health policy, it would be unfeasible to address all of them simultaneously. Therefore, it is important to underline that the present work is limited to the evaluation of the achievement of certain health functionings, namely: *infant mortality rate, under-five mortality rate, crude mortality rate *and *life expectancy*. These are intended as proxies of central health capabilities concerned with avoiding premature death. Accordingly, intergroup and inter-country differences in those parameters signal underlying health inequities that are not being addressed appropriately.

### Health Care Reform in Brazil

#### Pre-Reform Situation

Established under military rule (1964-1985), the old health care system in Brazil was characterized by a clear separation of functions between classical Public Health interventions (e.g. vaccination and disease surveillance), under the responsibility of the Ministry of Health, and individual care, which was regulated by the Ministry of Social Security. Whereas the Ministry of Health (MS, in Portuguese) saw a decline of its resources from 4.57% of the federal budget in 1961 to 1.38% in 1980, the Ministry of Social Security (MPAS, in Portuguese) gained ground, funded by compulsory social contributions of 8% of wages of formal sector employees. As a result, it accounted for more than 90% of all hospitalizations and outpatient consultations by 1975.

Despite its role as the core of the social insurance model, the delivery of care was, for the most part, left to private providers. In fact, the Constitution of 1967 determined that the State had to support the private sector, and that public provision could only be performed to *supplement *the role of private providers. Consequently, by the late 1970s, the health care arm of the National Institute of Social Security (INPS, in Portuguese) had contracted out with 2,300 of the 2,800 hospitals then established in Brazil [12]. Notwithstanding its increased reach of the urban masses, the system soon started to fall apart. Dismayed by low reimbursement rates and payment delays, private providers began in some cases reneging contracts with INPS, or gaming the system by either conducting unnecessary procedures that were better paid or up coding them. Fraud became the norm rather than the exception. Meanwhile, in the Public Health arena, malaria continued to scourge the Amazon region and several epidemic episodes of dengue and meningitis (in 1971 and 1974) swept the country, demonstrating the severe budgetary and technical shortfalls in the Ministry of Health. All of this was coupled with the persecution of any media enterprise that dared bring such news to the general public.

Alleging better management practices and modern facilities, private insurers thus emerged as substitutes of INPS in the formal labor market, filling the vacuum left by the crumbling public services. Many of those companies were vertically integrated, similarly to Health Maintenance Organizations (HMOs) in the United States. Not surprisingly, such developments were accompanied by an increased participation of foreign capital, both in the insurance and provision markets of the Brazilian health sector.

In opposition to the forces of privatization, an amalgam of social groups united behind a coalition commonly referred to as the Sanitary Reform Movement (SRM). Born out in the Departments of Preventive Medicine of Faculties of Medicine, most of which hosted by public universities, the SRM had a strong commitment to democratization, decentralization and de-medicalization (i.e. promotion of community-based primary care and opening of clinical practice to health care providers other than physicians). Supported by the Brazilian Catholic Church, academic institutions and popular social movements [13], its greatest challenge was to convince the middle and lower classes that a different approach was possible. To that end, several pilot projects were developed across the country as demonstrations of how the public provision of health care services could be better than what was being offered by INPS and the private sector.

#### Foundations of the Reform

Greatly influenced by Marxist theory and Foucault's social critique, the Sanitary Movement sought to establish a new model for the Brazilian health care system, which was closely related to the perspective of Social Medicine in vogue in Europe at that time, but adapted to the complex social and political realities faced in Brazil [13]. As such, it was fiercely egalitarian, supporting nothing but the full recognition by the State that health was a universal social right and that it must be provided equitably [14]. It also incorporated communitarian notions of local decision-making and resource allocation, thus the emphasis in decentralization. Moreover, it saw itself as a civil movement whose ultimate goal was to promote social justice and move beyond representational democracy into direct popular participation in policymaking.

Considering the disquieting centralist legacy of the military years and the growing dissatisfaction with the quality of care delivered by INPS and private providers, the SRM was able to build a broad coalition into the debates that eventually culminated in the National Constitutional Assembly of 1987-1988. Conversely, the struggle to create a publicly-funded national health system faced many opponents that thrived in the previous state of affairs. When competing interests within the coalition threatened its collapse [15], a compromise was forged with libertarian forces: a new public Unified Health System (SUS, in Portuguese) was to be created integrating all the public provision and regulation of health care under the auspices of the Ministry of Health. In exchange, the private system would not only continue to exist, but wealthy individuals would be able to deduct a large portion of their premiums and other private health care expenditures from their federal income tax.

#### Legal Framing

After more than two decades of dictatorship, the democratic Brazilian Constitutional Assembly finally defined health as a right in the National Constitution of 1988. According to Article 196: "*health is a right of all [citizens] and a duty of the State, guaranteed by social and economic policies aimed at reducing the risk of disease and providing universal and egalitarian access to actions and services for its promotion, protection and recovery*" (author's translation). Article 198 defined that health care was to be provided by a hierarchical, regionalized network of services that would constitute the Unified Health System. Its main principles would be decentralization, integral care and community participation [16]. Conversely, the Constitution did not specify how this right would be made operational. For instance, it did not make it clear how it would be financed, how it would be organized and managed, or what would be the duties and responsibilities of the different federative entities (Union, States and Municipalities).

Thus, a series of federal laws and ordinances were enacted in the following years to clarify those issues. Federal Law 8080 of 1990 defined as attributions of SUS: the delivery of preventive and curative services (including the provision of pharmaceutical drugs), the epidemiologic and sanitary surveillance, as well as the regulation of the entire health care system. It created the National Health Fund, from which all federal transfers to States and Municipalities would be made. It also regulated the participation of private providers within SUS and forbid foreign capital from participating in the domestic health care market. Federal Law 8142 of 1990 created the framework for social participation within SUS, by creating health councils in each sphere of government and mandating that half of its composition be assigned to patient representatives. The Basic Operational Norm of 1996 (NOB/1996) and the Operational Norm of Health Assistance of 2001 (NOAS/2001) specified the duties and responsibilities of each federative entity and how they would relate to one another. They also established the mechanisms of the intergovernmental transfer of resources and the conditions that States and Municipalities must meet in order to receive those funds.

In 2000, Constitutional Amendment 29 attempted to increase the level of public health expenditures by mandating minimal budgetary floors for Federal, State and Municipal health budgets. While State governments were required to assign 12% of their income to SUS, Municipal governments were required to assign 15%. Meanwhile, the Federal government was required to yearly increase their health budget by the nominal variation of the Gross Domestic Product (GDP). Nevertheless, the Amendment had a major shortcoming. It did not define what could be considered as health expenditure. As a result, several state and local governments included in their health budgets expenses that were previously assigned elsewhere (e.g. food stamps and health care for prisoners) in order to meet the new constitutional requirements, instead of incrementing Public Health activities or improving the delivery of care.

#### Health System Design

The health system in Brazil is mixed and segmented into two subsystems: one public and one private, with separate financing streams. The public subsystem has two segments: one provides free universal access (all citizens have the right), fully financed by public resources (general taxes and compulsory payroll taxes), called the *Unified Health System *(Sistema Único de Saúde - SUS, in Portuguese); the other is restricted to public employees (mainly military and high ranking civil servants), and it is financed by a traditional social insurance model based on contributions from public employees and the federal government.

The provision of services in SUS is usually carried out by public providers under the control of State or Municipal Health Departments, although it is possible to contract out to private providers. When doing so, laws give clear preference to other public non-governmental entities (e.g. universities) and not-for-profit Non-Governmental Organizations (NGOs). Alternatively, for-profit providers face severe restrictions to provide care under SUS. Another important aspect of current regulations is that regardless of who delivers the services, Health Departments are deemed co-responsible, and therefore liable, for any malpractice by contractors.

The private subsystem is also comprised of two segments, both of which benefit from some form of fiscal incentive: the first is known as the *supplementary system *and encompasses several modalities of health insurance. Participation is voluntary, and it is financed either with resources from employers and employees (the rates of contribution are freely negotiated between the parties) or exclusively by individual families. The second segment offers direct access to private providers through out-of-pocket payments [17]. It is worth noting that the population covered by the private subsystem also benefits from the public network through public health activities (e.g. vaccination campaigns), and some also use it for more complex or costly procedures not covered by their private health insurance policies.

Since 1999, individual health insurance policies are overseen by the National Agency for Supplementary Health (ANS, in Portuguese), which has standardized three types of benefit packages that insurance companies can offer. The first covers just outpatient care, the second inpatient care and emergency services, and the third covers all of the above. Each package has a required set of diagnostic and therapeutic procedures that have to be covered. Insurance companies are free to define their providers' network and pricing policies, but are forbidden from excluding anyone on the basis of pre-existing conditions (i.e. cream-skimming). They can, however, deny care for pre-existing conditions for up to two-years. Furthermore, every year they must request an authorization from ANS for any increase in premiums. The main caveat of this legislation is that group insurance policies (i.e. those directly negotiated by employers on behalf of their employees) are exempt from most of those requirements, resulting in much greater diversity of coverage.

### Health Care Reform in Colombia

#### Pre-Reform Situation

The old health care system in Colombia was a three-tiered system, comprised of a public sector, a social insurance sector and private insurance. The Public Sector was publicly provided within the National Health System (SNS, in Spanish) and financed through general taxes. It developed considerably between 1975 and 1984, when it experienced a large increase in the number of hospitals, health care centers and personnel. However, the fiscal crisis of 1982 reduced public health care expenditures from 8% of the national budget to less than 4% on subsequent years [18]. Besides being underfunded, the distribution of those scarce resources was based on historical averages and political pressures, thus favoring the developed regions of the country. In terms of coverage, it targeted those who did not participate in the formal labor market and could not afford to purchase private insurance [19]. It effectively reached 27% of the population with an additional 28% covered only partially, exhibiting significant regional differences [18].

The social insurance sector provided coverage to 15% of the population through the Colombian Social Security Institute (ICSS, in Spanish), for those employed by the formal private sector, and the Public Provision Funds (CPP, in Spanish) for the majority of civil servants. This was one of the lowest rates in Latin America and the rate of tax evasion in these segments was fairly high, as only 50% of those required to contribute to the system actually did so [19]. In addition, the armed forces and some public employees (e.g. public school teachers and employees of the state oil company), comprising 5% of the population, had their own social insurance schemes and network of providers. Finally, only 10% of the Colombian population could afford private health care. The rest of the population, about 15%, had no access to acceptable health services [18].

#### Foundations of the Reform

Colombia's health care reform was as much a product of international influence by actors such as the World Bank and the Pan American Health Organization as it was part of a modernizing agenda brought by the national executive branch. Designed and implement by an external "change team" [20] harbored at the Ministry of Social Protection (MSP), which incorporated the former Ministry of Health. This change team was composed mostly by academic economists, many of which had had training abroad. Their ideological stand closely followed modernization theories promoted by the World Bank and in vogue at that time, such as changing the role of the State in the social sector from provider of services to regulator; promoting the role of the private sector; increasing efficiency, and using mechanisms other than those historically used in the delivery of social services, such as targeting and demand subsidies [20]. By redesigning the health sector, it sought to overcome the policies of the previous decades, which, as mentioned above, had built a fragmented three-tiered system resulting in constrained access to health services for a large proportion of the population, operational inefficiencies at all levels of care, and poor service quality [21].

#### Legal Framing

The decentralization of the public health sector started with Decree 77 of 1987 and Law 10 of 1990. In 1993 it was further emphasized by Law 60 and culminated with the reform of the entire Social Security System of Colombia enacted by Law 100. The latter were greatly influenced by the Constitutional Reform of 1991, which strongly promoted the decentralization of public services to sub national levels and ended governmental monopoly over public services, including health care [22].

Law 100 changed the organization, financing and delivery of Colombia's health care system, mandated the creation of a new system for the financing and delivery of health care, allocating public funds directly to individuals instead of institutions [21], thereby changing classic social policy from supply-side to demand-side subsidies. It also established the legal basis for separating the delivery of services from the financing mechanism by mandating the separation of public hospitals from the administrative apparatus of local governments and their conversion into semi-public entities referred to as State Social Enterprises (ESEs, in Spanish), allegedly to grant them the financial and managerial autonomy necessary to prepare for competition with the private sector under the new health insurance scheme [21].

#### Health System Design

Law 100 models individual health care services differently from traditional Public Health functions. Whereas the latter are seen as public goods and as such to be funded by public funds, the former are seen as goods with intrinsic individual value, for which consumers would be willing to pay. As such, individual health care services were reorganized as to become part of a National Health Insurance (NHI) scheme under a market-driven framework, which incorporated principles of managed competition [23].

The most important mechanisms of managed competition introduced in Colombia health care model were: 1) the mandate that all workers in the formal sector participate of NHI; 2) the existence of one single collecting fund to each all resources flow to, called the National Fund of Security and Guarantees (FOSYGA, in Spanish); 3) the establishment of a new payment mechanism to insurers, through a risk-adjusted capitation system; and 4) the definition of a standardized package of benefits to be offered to all insurance beneficiaries, called the Mandatory Health Plan (POS, in Spanish) [23].

Alleging resource constraints, the NHI was in effect designed as a two-tiered system, composed of the Contributory Regime (CR) and the Subsidized Regime (SR). The CR included all formal sector employees or independent workers with ability to pay who were already enrolled in some form of either private or public insurance, extending coverage to their families. Formal workers were set to contribute an equivalent to 12% of their salary, of which 4% was to be paid by the employee and 8% by the employer. Independent workers would pay the full 12% starting from a floor of 2 minimum wages. The SR targeted the poor and indigent population by providing subsidies to the insurance premium from specific public resources and contributions from the CR. For instance, a percentage point from payroll taxes was to be channeled to the SR. Tax revenues from several sources and social investment transfers to municipalities were also to be earmarked for health. Among these were new resources from oil revenues and matching funds by the national government to FOSYGA, the solidarity fund [21].

As resources became available, expansion in insurance coverage for the population eligible for subsidies would be accompanied by gradual additions to the benefits package. It was expected that both regimes would have identical coverage of benefits by 2000, so that a single universal health insurance system could be implemented throughout the nation [21]. However, by 2002 the SR beneficiaries were entitled to only 70% (or less, depending on where they lived) of the standard benefits package enjoyed by CR enrollees [23].

The delivery of services was to be carried out by both public and private providers, which would compete among themselves under the watchful eyes of regulatory authorities. Nevertheless, due to funding constraints and cumbersome administrative structures, public hospitals have lost the upper hand to private providers. Consequently, 14 years later, most of the provision of health care services in Colombia has been *de facto *privatized.

## Methods

### Study Design

Although several different methods have been suggested to evaluate the performance of health systems [24-26], and of health care reforms more specifically [27], the one most consistent with cross-country comparisons of inequities in health capabilities is the measurement of shortfall inequalities [3]. At the national level, such measures can assess quantitatively how much a given society has realized its health potential and how much remains unrealized, because they compare the actual achievement of a given health system with the optimal average value of a given reference group [3]. If the objective of the study is to understand domestic inequities than the reference group shall be the segment of the population who presents the highest achievement. For international comparisons, the best performing country for a given indicator shall be used as the reference. This allows for both methodological flexibility and consistency. For those reasons, this is the standard adopted by this work.

### Data

The data for outcome variables was drawn from BADEINSO, a cross-national database of social indicators produced by the Economic Commission for Latin America and the Caribbean (ECLAC) [28]. It contains 23 different indicators, for 35 countries (including semi-independent territories), covering the period of 1960-2007. The data for the control variables were drawn from WDI (World Development Indicators), a cross-national database produced by the World Bank [1]. It contains more than 800 economic and socio-economic indicators for 209 countries, covering the same time-period as that of BADEINSO. According to ECLAC and the World Bank, data included in those databases conform as much as possible to the United Nations System of National Accounts (SNA) and the methods of specialized U.N. agencies (e.g. WHO and UNICEF). Despite their breadth, a crucial issue with both databases is the prevalence of insidious gaps in the data, most noticeably for series earlier than 1990, which posed some statistical challenges and limited the scope of this analysis.

### Measures

The four primary outcome measures (dependent variables) in this study were crude mortality rate (CMR - the number of deaths in a given year divided by 1,000 inhabitants), infant mortality rate (IMR - the number of deaths of children less than 1 year old per 1,000 live births), under-five mortality rate (U5MR - the number of deaths of children less than 5 years old per 1,000 live births), and life expectancy at birth (LEXP - the expected average life-span measured in years). The main control variables were gross domestic product (GDP) per capita, converted to constant 2000 U.S. dollars), fertility rate (number of births per woman), population growth (annual percentage of growth) and rural population (percentage of the population living in rural areas). The first step in this analysis was to decide what would be time interval used to evaluate the time series. Considering several gaps in the data, a 5-year interval was chosen, covering the period 1960-2005. Subsequently, when data for any specific time point was missing, the respective value was estimated from a five-year average of adjacent years. As a result, a total of 10 observations for each country were obtained for each variable.

The second set of outcome variables (ABSSI_CMR, ABSSI_IMR, ABSSI_U5MR, and ABSSI_LEXP) was calculated by comparing the actual value of the original indicators for each of the two countries against the optimal average value for Latin America and the Caribbean in order to obtain the absolute shortfall inequality for each country in each given time period, using the following equation: ***SI_x _= | X_optimal _- X_actual _| ***, where *X *represents the value of the indicator being analyzed in each given year of the time series.

Finally, in order to evaluate the reform, two dummy variables were created. The first one was created to represent whether any given time point was either before or after (pre/post) the health care reforms. The second one was created to represent how many years had elapsed since the reforms. The years in which the major legislative changes were enacted (1988 and 1993, respectively for Brazil and Colombia) were defined as the break points for each country time series.

### Graphical Analysis

For each outcome variable, the time-series values for Brazil and Colombia were plotted against the optimal average value for the region (Latin America and the Caribbean).

### Statistical Analysis

Overall characteristics of each variable were assessed by univariate analysis. Subsequently, control variables were tested for correlation. High values (greater than 95%) were found among them. As a result, GDP per capita was chosen as the sole control (independent) variable in the multivariate models in order to avoid multicollinearity. Bivariate analysis examined unadjusted relationships between the outcome variables and the overall time trend as well as the reform. Bivariate correlations were analyzed with the t-test. The null hypothesis in all the tests was that the health care reforms had no impact in the reduction of health inequities, whether analyzed separately for each country or when compared to the optimal regional average value (in terms of the shortfall inequality).

Multivariate analyses were performed using ordinary least square regression. Separate sets of models were used for each country (Brazil and Colombia), for each outcome (dependent) variable. To test for a change point difference (i.e. to see the impact of the reforms in each outcome variable), two approaches were used. The first approach was to fit models which included the year span and the second reform dummy, in order to assess the impact of the reform while controlling for the overall time trend. Subsequently, this initial model served as the basis for another model, which included the variable GDP per capita to also control for economic development. The second approach was to perform predictive Chow tests in all of the above models, in order to test for structural change by evaluating whether the coefficients in the regression model were the same in the separate subsamples (pre/post reform).

As expected by the nature of time-series analysis, two of the dependent variables (IMR and U5MR) presented second-order lag influence in the linear regressions of the Brazilian data using the first set of models (time trend + reform). Accordingly, new estimates were obtained by correcting for the autoregressive parameters. No other adjustments were made. Multivariate linear regressions were validated using adjusted-R^2 ^and partial-F tests. Two-tailed p-values are reported for all analyses. All statistical analyses were performed using SAS^® ^and Microsoft Excel^®^.

## Results

### Crude Mortality Rate

**Figure 1:**
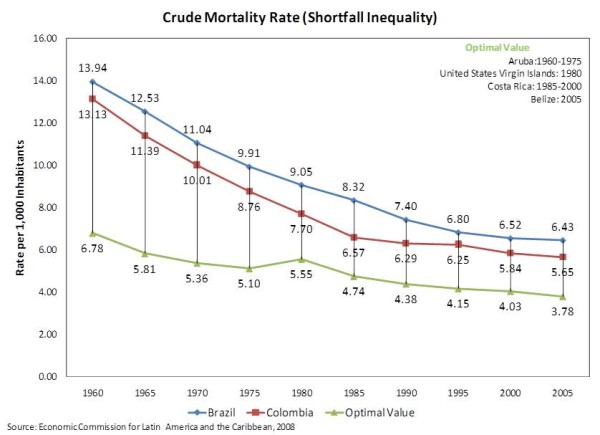
**Crude Mortality Rate (Shortfall Inequality) **Time-Series trend data of Crude Mortality Rate for Brazil and Colombia.

The graphic analysis of the time-series trend data for crude mortality rate shown in Figure 1 demonstrates that both countries succeeded in reducing their respective rates, but that the reduction of the shortfall inequality fluctuated considerably and slowed down after 1985 for Colombia and 1990 for Brazil.

The bivariate analyses of the baseline values of crude mortality rate showed a persistent, and similar, trend of reduction in this rate for both Brazil (-*0.16893 *per year) and Colombia (-*0.16183 *per year). The predictive Chow test also confirmed that a break in the time-series seemed in fact to have happened at the time each one of the reforms was enacted. Indeed, the average crude mortality rates for the post-reform periods were *4.01080 *units smaller for Brazil and *3.20810 *units smaller for Colombia.

However, the multivariate analyses of the baseline values pointed out in the opposite direction. Indeed, when the effect of the reform was controlled for the underlying time trend, for each year that followed the enactment of the reforms, the pace of reduction of the crude mortality rate was either slowed down in the case of Brazil, or nearly reversed in the case of Colombia. Both results were statistically significant at *p = 0.0004 *and *p = 0.0059*, respectively for Brazil and Colombia. The pattern persisted even when the influence of GDP per capita was factored in, as shown in Table 1.

**Table 1:** Crude Mortality Rate: Baseline Values

	Brazil				Colombia			
	***Parameter Estimate***	***p-value***	***Adjusted R^2^***	***Predictive Chow***	***Parameter Estimate***	***p-value***	***Adjusted R^2^***	***Predictive Chow***

**Bivariate Analyses**								

Time Trend	-0.1686	< .0001	0.9375	0.0434	-0.1618	< .0001	0.8797	0.0193

Reform	-4.0108	0.0067	0.6217	0.9872	-3.2081	0.0680	0.2770	0.9701

**Multivariate Analyses**								

*1st model*			0.9895	0.9620			0.9567	0.3734

Time Trend	-0.2244	< .0001			-0.2134	< .0001		

*plus *Reform Years	0.1655	0.0004			0.2545	0.0059		

*2nd model*			0.9882	0.9818			0.9576	0.5652

Time Trend	-0.2107	0.0005			-0.3174	0.0180		

*plus *Reform Years	0.1533	0.0068			0.3059	0.0089		

*plus *GDP per capita	-0.0002	0.6577			0.0032	0.3240		

Likewise, Table 2 shows a pattern similar to what was observed with the baseline values. While the bivariate analyses appear to demonstrate an important effect of both the time trends and the reforms in reducing the shortfall inequality in the crude mortality rate, the multivariate analyses that control for the underlying time trend pointed out in the opposite direction: that the years after the reforms either reduced the pace of the improvement in the crude mortality rate or upturned previous gains. Once more, the effect on Colombia seems to have been more deleterious than in Brazil.

**Table 2:** Crude Mortality Rate: Shortfall Inequality

	Brazil				Colombia			
	***Parameter Estimate***	***p-value***	***Adjusted R^2^***	***Predictive Chow***	***Parameter Estimate***	***p-value***	***Adjusted R^2^***	***Predictive Chow***

**Bivariate Analyses**								

Time Trend	-0.1099	< .0001	0.8874	0.0835	-0.1031	0.0005	0.7728	0.0356

Reform	-2.5392	0.0129	0.5043	0.9988	-1.8062	0.1415	0.1557	0.9928

**Multivariate Analyses**								

*1st model*			0.9725	0.8765			0.9012	0.4298

Time Trend	-0.1578	< .0001			-0.1484	< .0001		

*plus *Reform Years	0.1424	0.0014			0.2242	0.0118		

*2nd model*			0.9871	0.6003			0.8868	0.5130

Time Trend	-0.0963	0.0046			-0.1838	0.1406		

*plus *Reform Years	0.0877	0.0161			0.2416	0.0341		

*plus *GDP per capita	-0.0008	0.0242			0.0011	0.7511		

### Infant Mortality Rate

**Figure 2:**
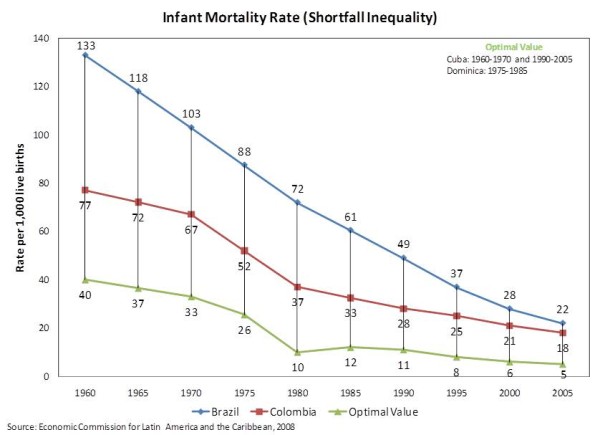
**Infant Mortality Rate (Shortfall Inequality) **Time-Series trend data of Infant Mortality Rate for Brazil and Colombia.

The graphic analysis of the time-series trend data for infant mortality rate shown in Figure 2 demonstrates that both countries succeeded in reducing their respective rates, but that the reduction of the shortfall inequality was proportionally more intense in Brazil than in Colombia, although Colombia has always maintained a lower rate.

The bivariate analyses of the baseline values of infant mortality rate showed a much steeper trend of reduction in this rate than in the crude mortality rate, both for both Brazil (-*2.52848 *per year) and Colombia (-*1.42364 *per year). The predictive Chow test suggested that a break in the time-series seemed to have happened at the time the reform was enacted in Brazil but not so in Colombia. Nevertheless, the average infant mortality rates for the post-reform periods were both substantially smaller: -*61.66667 *in Brazil and *-30.88095 *in Colombia.

The multivariate analyses of the baseline values pointed out that such gain might not have been due to the reforms. Instead, the original time trend seems to have remained the main driver, even after the reforms were enacted. In fact, when that effect was controlled for, each subsequent year after the reforms saw the slowdown of the reduction of the infant mortality rate in both countries. Nonetheless, the downturn in Brazil seems to have been less pronounced than in Colombia (*1.19610 *vs. *1.54147*). Both results were statistically significant at *p < 0.0001 *and *p = 0.0164*, respectively. As shown in Table 3 such pattern did not change substantively when GDP per capita was also controlled for.

**Table 3:** Infant Mortality Rate: Baseline Values

	Brazil				Colombia			
	***Parameter Estimate***	***p-value***	***Adjusted R^2^***	***Predictive Chow***	***Parameter Estimate***	***p-value***	***Adjusted R^2^***	***Predictive Chow***

**Bivariate Analyses**								

Time Trend	-2.5285	< .0001	0.9861	0.0041	-1.4236	< .0001	0.9298	0.1073

Reform	-61.6667	0.0032	0.6441	0.9573	-30.8810	0.0339	0.0339	0.9938

**Multivariate Analyses**								

*1st model*			0.9985	0.4627			0.9667	0.7786

Time Trend	-2.9222	< .0001			-1.7357	< .0001		

*plus *Reform Years	1.1961	< .0001			1.5415	0.0164		

*2nd model*			0.9987	0.3530			0.9624	0.5453

Time Trend	-2.7148	< .0001			-1.3746	0.1347		

*plus *Reform Years	0.9895	0.0018			1.3631	0.0805		

*plus *GDP per capita	-0.0026	0.1978			-0.0112	0.6608		

When shortfall inequalities were considered (Table 4), any positive effect the reforms might have had in reducing the infant mortality rate seem to have been further attenuated. For instance, predictive Chow tests were unable to distinguish any statistically significant break point in the overall time trend, meaning the reforms did not seem to have had any impact in the reduction of the shortfall inequalities throughout the analyzed period, not even in Brazil where the predictive Chow test was statistically significant for the time trend in the baseline values. Similarly, the multivariate analyses that control for the underlying time trend and GDP per capita indicated that the reforms did not alter the processes that had already been set into motion before them.

**Table 4:** Infant Mortality Rate: Shortfall Inequality

	Brazil				Colombia			
	***Parameter Estimate***	***p-value***	***Adjusted R^2^***	***Predictive Chow***	***Parameter Estimate***	***p-value***	***Adjusted R^2^***	***Predictive Chow***

**Bivariate Analyses**								

Time Trend	-1.6861	< .0001	0.9861	0.8711	-0.5812	< .0001	0.9515	0.3601

Reform	-43.0000	0.0012	0.7159	0.8860	-13.2143	0.0215	0.4416	0.9867

**Multivariate Analyses**								

*1st model*			0.9847	0.9320			0.9654	0.9120

Time Trend	-1.7376	< .0001			-0.6653	< .0001		

*plus *Reform Years	0.1528	0.6338			0.4154	0.0792		

*2nd model*			0.9894	0.9859			0.9606	0.9508

Time Trend	-2.2924	0.0002			-0.7879	0.0539		

*plus *Reform Years	0.6455	0.1165			0.4759	0.1271		

*plus *GDP per capita	0.0069	0.0886			0.0038	0.7183		

### Under-Five Mortality Rate

**Figure 3:**
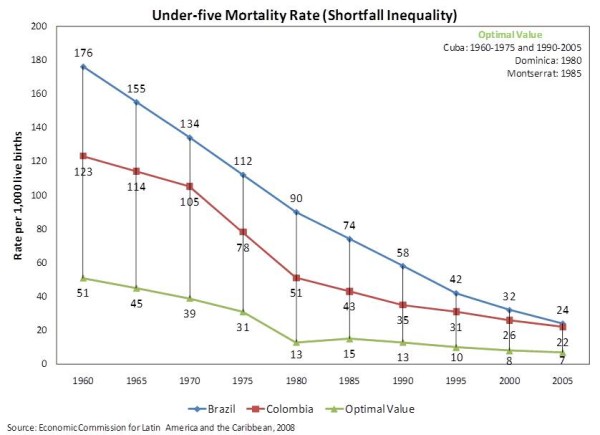
**Under-Five Mortality Rate (Shortfall Inequality) **Time-Series trend data of Under-Five Mortality Rate for Brazil and Colombia.

The graphic analysis of the time-series trend data for under-five mortality rate shown in Figure 3 exhibits a similar layout as the one for infant mortality rate. Considering that under-five mortality rate encompasses the infant mortality rate, such outlook is hardly a surprise. On the other hand, what was unanticipated was the fact that the gap between Brazil and Colombia seems to be closing even faster than in the case of infant mortality rate. If the current outline holds, Brazil may soon have a lower rate than Colombia.

The bivariate analyses of the baseline values of under-five mortality rate exhibited a slightly greater trend of reduction in this rate than in the infant mortality rate, both for both Brazil (*-3.47515 per year*) and Colombia (-2*.46303 per year*). Results from the predictive Chow test once again implied the presence of a significant break in the Brazilian time-series but not the Colombian. Nonetheless, the average under-five mortality rates for the post-reform periods were substantially reduced in both countries: -*84.50000 *in Brazil and *-52.09524 *in Colombia.

The multivariate analyses of the baseline values pointed out that such improvement was slowed down in the years after the reform, to the point of nearly reversing the trend in Colombia (time trend: *-3.10686 */reform years: *3.18061*). Both results were statistically significant at *p < 0.0001 *and *p = 0.0095*, respectively. As shown in Table 5 such pattern did not change substantively when controlled for GDP per capita.

**Table 5:** Under-Five Mortality Rate: Baseline Values

	Brazil				Colombia			
	***Parameter Estimate***	***p-value***	***Adjusted R^2^***	***Predictive Chow***	***Parameter Estimate***	***p-value***	***Adjusted R^2^***	***Predictive Chow***

**Bivariate Analyses**								

Time Trend	-3.4752	< .0001	0.9806	0.0026	-2.4630	< .0001	0.9107	0.0711

Reform	-84.5000	0.0035	0.6357	0.9691	-52.0952	0.0429	0.3467	

**Multivariate Analyses**								

*1st model*			0.9984	0.5178			0.9633	0.7484

Time Trend	-4.1248	< .0001			-3.1069	< .0001		

*plus *Reform Years	1.9478	< .0001			3.1806	0.0095		

*2nd model*			0.9987	0.3387			0.9587	0.5262

Time Trend	-3.8282	< .0001			-2.4524	0.1433		

*plus *Reform Years	1.6747	0.0006			2.8573	0.0531		

*plus *GDP per capita	-0.0037	0.1799			-0.0203	0.6640		

When shortfall inequalities were evaluated (Table 6), the bivariate analyses pointed out that the positive impact of the reforms were smaller than what was originally suggested by the analysis of the baseline values. The multivariate analyses only made that distinction clearer. In the context of Brazil, every year after the reform seem to have marginally negated the effect of the underlying time trend (an increase of *0.57245 *in the inequality vs. a decrease of *2.63532*), although not statistically significant. More startling was the impact in Colombia, where reform years increased the shortfall inequality by *1.66040 *a year (*p = 0.0134*), all but erasing the progress represented by the underlying trend of *-1.76641 *(*p < .0001*). The introduction of GDP per capita into the model produced no significant changes in the analysis.

**Table 6:** Under-Five Mortality Rate: Shortfall Inequality

	Brazil				Colombia			
	***Parameter Estimate***	***p-value***	***Adjusted R^2^***	***Predictive Chow***	***Parameter Estimate***	***p-value***	***Adjusted R^2^***	***Predictive Chow***

**Bivariate Analyses**								

Time Trend	-2.4424	< .0001	0.9901	0.4296	-1.4303	< .0001	0.9228	0.1012

Reform	-61.6667	0.0015	0.7022	0.9265	-30.8571	0.0361	0.3715	0.9972

**Multivariate Analyses**								

*1st model*			0.9924	0.8195			0.9652	0.8098

Time Trend	-2.6353	< .0001			-1.7664	< .0001		

*plus *Reform Years	0.5725	0.1100			1.6604	0.0134		

*2nd model*			0.9942	0.9371			0.9599	0.7290

Time Trend	-3.1588	< .0001			-1.5472	0.1109		

*plus *Reform Years	1.0373	0.0330			1.5521	0.0614		

*plus *GDP per capita	0.0065	0.1232			-0.0068	0.7969		

### Life Expectancy

**Figure 4:**
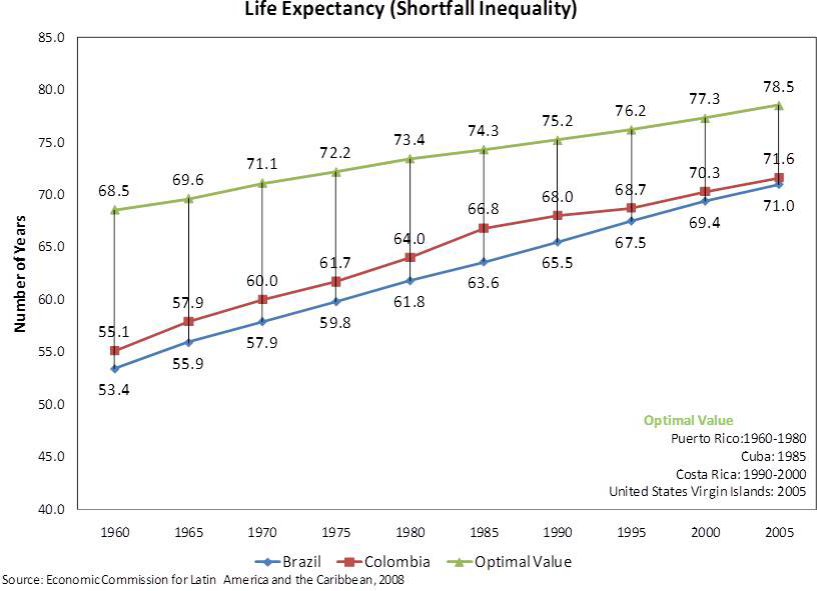
**Life Expectancy (Shortfall Inequality) **Time-Series trend data of Life Expectancy for Brazil and Colombia.

The graphic analysis of the time-series trend data for life expectancy shown in Figure 4 demonstrates that both countries have progressively increased the average life-span of their populations. Also perceptible is the apparent leveling off of the rate of improvement in Colombia since 1990, in the 68-71 range. On the other hand, Brazil seems to be maintaining a fairly linear rate of improvement and might soon outpace Colombia. Still, both countries loom distant from the optimal value in Latin America and the Caribbean.

The bivariate analyses of the baseline values of life expectancy confirm the expectations geared from Figure 4. In other words, both countries have consistently improved the life expectancies of their populations, although Brazil seems to have advanced at a somewhat higher rate than Colombia (*0.38764 *year gained per calendar year vs. *0.36424*), which justifies their near tie in 2005. Accordingly, the predictive Chow test for the time trend was statistically significant only for Colombia, meaning that the break point represented by the reforms was not observed in Brazil. Unfortunately, that was not necessarily a positive outcome, quite the opposite.

As the multivariate analyses showed in Table 7 the rate of the improvement in life expectancy was more than halved (*-0.23368 *per year*; p = 0.0090*) in the years after the reform in Colombia. Conversely, such period in Brazil produce only a non-statistically-significant (*p = 0.0678*) reduction of *-0.03861 *per year. No significant additional effect was observed when GDP per capita was included in the model.

**Table 7:** Life Expectancy: Baseline Values

	Brazil				Colombia			
	***Parameter Estimate***	***p-value***	***Adjusted R^2^***	***Predictive Chow***	***Parameter Estimate***	***p-value***	***Adjusted R^2^***	***Predictive Chow***

**Bivariate Analyses**								

Time Trend	0.3876	< .0001	0.9985	0.4545	0.3642	< .0001	0.9768	0.0135

Reform	9.6167	0.0020	0.6797	0.8684	8.2714	0.0196	0.4535	0.9528

**Multivariate Analyses**								

*1st model*			0.9990	0.7540			0.9906	0.1989

Time Trend	0.4007	< .0001			0.4116	< .0001		

*plus *Reform Years	-0.0386	0.0678			-0.2337	0.0090		

*2nd model*			0.9988	0.8433			0.9937	0.1805

Time Trend	0.4012	< .0001			0.5810	0.0004		

*plus *Reform Years	-0.0391	0.1927			-0.3174	0.0030		

*plus *GDP per capita	0.0000	0.9792			-0.0053	0.0782		

When shortfall inequalities were evaluated (Table 8), the bivariate analyses showed that the negative impact seen in the years after the reform was even more dramatic for Colombia. Furthermore, the multivariate analyses demonstrated that instead of getting smaller, shortfall inequalities in life expectancy actually grew an average of *0.21585 *per year, overriding the underlying reductionist time trend of *-0.19078 *per year. In the case of Brazil, no statistically significant change was observed in the multivariate analysis, except for the time trend, which was significant in both multivariate models (*p < .0001*). The introduction of GDP per capita into the model only produced significant changes in the analysis of the Colombian time-series, where it also acted against the reduction of the shortfall inequalities in life expectancy.

**Table 8:** Life Expectancy: Shortfall Inequality

	Brazil				Colombia			
	***Parameter Estimate***	***p-value***	***Adjusted R^2^***	***Predictive Chow***	***Parameter Estimate***	***p-value***	***Adjusted R^2^***	***Predictive Chow***

**Bivariate Analyses**								

Time Trend	-0.1705	< .0001	0.9930	0.4811	-0.1471	< .0001	0.8923	0.0259

Reform	-4.3358	0.0012	0.7164	0.8452	-2.9795	0.0586	0.3000	0.9589

**Multivariate Analyses**								

*1st model*			0.9923	0.1140			0.9599	0.2800

Time Trend	-0.1747	< .0001			-0.1908	< .0001		

*plus *Reform Years	0.0126	0.5820			0.2159	0.0066		

*2nd model*			0.9942	0.1891			0.9808	0.1314

Time Trend	-0.2115	< .0001			-0.3634	0.0009		

*plus *Reform Years	0.0452	0.1356			0.3011	0.0008		

*plus *GDP per capita	0.0005	0.1214			0.0054	0.0262		

## Discussion

Many countries in Latin America have undergone substantial reforms of their health care systems in the last twenty years. Measuring their impact on health outcomes is crucial to understand how effective they have been in achieving their stated goals [29-31] of promoting better access to health care services, financial security and reducing health inequities. To this date, most evaluations [23,31,32] of those reforms have been limited to single country analyses based on national cross-sectional data. The disadvantage of such methodology is that it does not adequately control for existing underlying time trends present in the variables of interest at the time the reforms were enacted. Furthermore, comparisons between countries are difficult to assess based on those individual studies since they often entail different variable definitions, different time periods and survey methods.

The present work thus attempted to redress those issues by utilizing internationally standardized time series data for Brazil and Colombia, covering the period from 1960 to 2005. Another innovation introduced by this study was that it is the first to compare the achievements in health functioning for Brazil and Colombia within the context of the *health capability paradigm *[3]. As a result, not only did it analyze their individual performances in the four health outcomes of interest (*crude mortality rate, infant mortality rate, under-five mortality rate *and *life expectancy*), but also assessed their success in reducing the shortfall inequalities in those variables when measured against the corresponding optimal values in Latin America and the Caribbean.

The results of the analyses of the baseline values of each country showed that while both of them succeeded in improving those indicators throughout the time period of the analysis, the impact of their health care reforms was much less tangible. In fact, in most cases they were not able to alter the underlying time trend already in place when the reforms were enacted. However, when they did influence the outcomes, the outline was inevitably negative. In other words, *the years after the reform often saw the deceleration of the pace of improvements in both countries for all the variables analyzed*. This effect was even more pronounced in Colombia, where some of the previous gains in the under-five mortality rate and the crude mortality rate were reversed.

The subsequent analyses of the shortfall inequalities further aggravated the perception that the health care reforms of Brazil and Colombia did not contribute to the reduction of heath inequities. When controlling for the underlying time trend, neither reform produced a statistically significant positive contribution to any of the variables studied. On the contrary, their respective impacts were often counterproductive. Consistent with the above results, Colombia fared once again worse than Brazil. In fact, both in life expectancy and crude mortality rate the average measured effect of the reform years overturned the historical time trend and effectively increased shortfall inequalities among those variables. Also worth noticing is the fact that GDP per capita, used as a control in the second model of the multivariate linear regressions, only had a statistically significant impact in two of them, namely: shortfall inequality of crude mortality rate in Brazil (*p = 0.0242*) and shortfall inequality of life expectancy in Colombia (*p = 0.0262*). When it did so, its influence was to increase the inequities.

Given the public praise received by both reforms from domestic [22,31,33,34] and international [8,17,35] observers in recent years, the findings of this study seem counterintuitive. How can this be true? First, most previous studies did not explicitly control for the effect of the historical time trend on results measured after the reform. Second, when they did control, it was within a differences-in-differences design for pooled cross-sectional data, often with just one pre-reform time period and one post-reform time period. Third, nearly all of them used national data, some of which might not have been adjusted to international standards, making cross-country comparisons much more difficult. Fourth, none of them measured the reforms of Brazil and Colombia against an independent third-party reference value in order to assess the effective reduction of the gap between their current achievements and the optimal international average (i.e. shortfall inequality) for the region.

Still, the present work has some limitations that must be acknowledged. First, the scarcity of internationally standardized data for health outcomes, especially for time points before 1990, severely restricted the diversity of outcomes that could be analyzed, thus forcefully limiting the scope of this study to the four health outcomes mentioned earlier. Second, because of such scarcity, a 5-year interval between data points had to be observed rather than an annual or semester basis, thereby increasing the influence of each individual data point in the overall fit of the regressions. Third, because of gaps in the dataset a few data points had to be estimated from 5-year averages, increasing standard errors and reducing the likelihood of statistically significant results. Fourth, the cut point of this study was set for 2005, when the Brazilian health care reform had been established for just over 15 years while the Colombian reform had little more than 10 years, both of which might be too short to comprehensively evaluate their long-term effects. Fifth, despite the efforts of ECLAC and the World Bank to standardize the data across countries, differences in methods and definitions might still account for some of the variability observed in the results of this study. Sixth, the attempt to control for the underlying historical trends assumes that such trends would remain constant in the years after the reforms were enacted, which does not account for countries' susceptibility to unforeseen economic shocks and social upheavals. Seventh, in the absence of health care reforms, it is possible that historical trends might not have remained constant throughout the time-series, given the potential plateau effect related to the ability to maintain constant changes as levels of performance rise, such as when outcomes improve.

In spite of such limitations, the high adjusted-R^2 ^of most of the models and their consistent pattern across the four studied variables suggest that the impact of the health care reforms in Brazil and Colombia was not nearly as positive as expected. Why? Some of the answers might lie outside the realm of the health care system while others could be nested in the heart of the design of each health care reform.

Within the realm of health care reforms, some features are likely culprits. For instance, in both Brazil and Colombia insufficient funding is likely to have slowed the implementation of new services that could have helped alleviated suffering populations. By extension, the overt emphasis on decentralization might have led to the disruption of previously functional vertical programs aimed at child survival, which in turn might have adversely effected the infant and under-five mortality rates. In the case of Colombia, the explicit tiering of the social insurance model into a contributory regime and a subsidized regime with distinct packages of benefits might have crystallized health inequities due to uneven access to care. Furthermore, such an arrangement is inherently unfair according to the health capability paradigm [3], as it denies equal access to high-quality care, an essential requirement in order to secure the achievement of central health capabilities. Moreover, the latest ruling of the Colombian Constitutional Court stroke down the two-tiered system, considering it unconstitutional and demanding a complete overhaul by 2010. On the other hand, although the Brazilian public health care system (SUS) is nominally universal and every citizen is entitled to comprehensive care, high regional inequities in access remain [36], thereby perpetuating long-lasting health disparities, which in turn hinder progress in the outcomes analyzed by this study. For instance, a recent study [37] presents a thorough analysis of sub-national inequalities in Brazil for under-five mortality rates and neonatal mortality rates which demonstrates that, in general, the Brazilian health care reform has been much more effective in improving rates in the better off than the worst off municipalities of the country. In many cases, this has led to widening poor/rich gaps.

Outside the health sector, both countries have endured imposing challenges. In the economic arena, the currency crises in Russia, Mexico and Southeast Asia that happened in the 1990's severely battered the overall economic stability of both countries. In this regard, Colombia seems to have suffered the most, since its economy is more dependent on the external market than the Brazilian economy. The graphs seem to corroborate this hypothesis, as a drastic slowdown in the rate of reduction of infant and under-five mortality rate starting at 1990 is unmistakable for Colombia but not as meaningful for Brazil. In addition, Colombia has been embattled in a fierce fight against domestic insurgent groups, particularly after 1990, with likely adverse effects in its crude mortality rate and life expectancy. Likewise, Brazil has also had to grasp with growing urban violence since 1995, possibly resulting in a slowdown of any progress in the reduction of its crude mortality rate. Nevertheless, no significant impact of this problem has yet been noticed in Brazil's life expectancy, perhaps because its effect might take longer to accrue. Another relevant aspect of the social and political scenario of both countries that is likely to be thwarting progress in health capabilities is the high level of income inequalities, as described by Gini coefficients of 0.569 for Brazil (2004) and 0.563 for Colombia (2003).

## Conclusions

This study has found that the health care reforms of the late 1980s in Brazil and early 1990s in Colombia do not seem to have had a positive impact in improving the crude mortality rate, the infant mortality rate, the under-five mortality rate or life expectancy when controlled for the underlying historical time trend. On the contrary, for all studied variables, the progress that otherwise would have been expected appeared to have been deterred in the years after the reforms were enacted. In the case of Colombia, such deterrence all but overturned previous achievements. The reasons for such failures are likely to be related both to the specific design of the reforms themselves, as well as external factors. Underfunding, unbridled decentralization, and inequitable access to care seem to be the main drivers among the former. Concerning the latter, the major impediments might have come from economic adversities, high levels of rural and urban violence, along with entrenched income inequalities.

Fortunately, governments in both countries seem to have awakened from those problems and have recently proposed measures to correct the flaws in the design and implementation of their respective health care reforms [36,38]. Nevertheless, further research needs to be done to measure the specific contribution of each one of the factors mentioned above in order to assist in the development of evidence-based health policy. Moreover, a systematic approach of monitoring and evaluation, especially standardized data collection, is of paramount importance. Neither individual countries nor international organizations can shy away from such commitments anymore. As Julio Frenk once noted: "to reform we need to inform, otherwise one is likely to deform" [39].

Likewise, in order to be able to inform, policymakers need evidence of what type of policy works best in each circumstance and what to expect from them. The aim of the present work was precisely to help fill this gap. I believe the results of this study can shed some light into the shady corners of health care reforms and further advance the quest for equity in Latin America.

## Competing interests

I declare no competing interests.

## Author's information

Although I am presently a government official at the Ministry of Health of Brazil, the research presented in this article was conducted before my governmental appointment, during my Master of Public Health at Yale University. The views and standpoints presented here are solely mine, and do not necessarily represent those of, or are sanctioned by, either the Ministry of Health of Brazil or Yale University.
